# Single-cell transcriptomic analysis reveals that the circRNA circGCLM promotes tumorigenesis and confers cisplatin resistance in NSCLC through the miR-505-3p/ERBB4 axis

**DOI:** 10.1016/j.tranon.2026.102759

**Published:** 2026-04-07

**Authors:** Yirou Ma, Baiqing Huang, Haiyang Wang, Mengchao Xue, Yiyang Liu, Yibo Jin, Chunping Wang, Shaorui Gu, Yongxin Zhou

**Affiliations:** Department of Thoracic and Cardiovascular Surgery, Tongji Hospital of Tongji University, School of Medicine, Tongji University, Shanghai, China

**Keywords:** CircGCLM, miR-505-3p, ERBB4, NSCLC, DDP resistance

## Abstract

**Background:**

The circular RNAs (circRNAs) have been implicated in chemoresistance, yet the specific roles of individual circRNAs in non-small cell lung cancer (NSCLC) remain largely unexplored. Here, we identified circGCLM as a novel driver of NSCLC tumorigenesis and cisplatin (DDP) resistance, and investigated its molecular mechanism of action.

**Methods:**

CircGCLM expression was examined in DDP-resistant and parental NSCLC cells. Loss-of-function studies were conducted to evaluate the effects on cell proliferation, apoptosis, and DDP sensitivity. The circGCLM/miR-505-3p/ERBB4 axis was validated using dual-luciferase reporter assays, RNA immunoprecipitation (RIP), and rescue experiments. ERBB4 expression dynamics was uncovered by single-cell transcriptomic analysis.

**Results:**

CircGCLM was markedly upregulated in DDP-resistant NSCLC cells compared to their parental cells. Functional loss-of-function studies demonstrated that circGCLM knockdown suppressed proliferation, restored DDP sensitivity, and promoted apoptosis in these resistant cells. Mechanistically, circGCLM acted as a competitive endogenous RNA (ceRNA) for microRNA-505-3p (miR-505-3p), which led to the derepression and consequent upregulation of its target, erb-b2 receptor tyrosine kinase 4 (ERBB4). Thus, the oncogenic functions of circGCLM, including the promotion of DDP resistance and other malignant phenotypes, were mediated through the miR-505-3p/ERBB4 axis.

**Conclusions:**

In summary, our findings established that circGCLM contributed to DDP resistance in NSCLC, at least in part, by sponging miR-505-3p to derepress ERBB4, highlighting a potential therapeutic target for overcoming chemoresistance.

## Highlights

• CircGCLM is identified as a critical determinant of DDP resistance in NSCLC.
• A novel CircGCLM / miR-505-3p / ERBB4 regulatory axis is defined in conferring DDP resistance.
• ERBB4 expression is profiled in NSCLC at single-cell resolution.

## Graphical abstract

**Figure:**
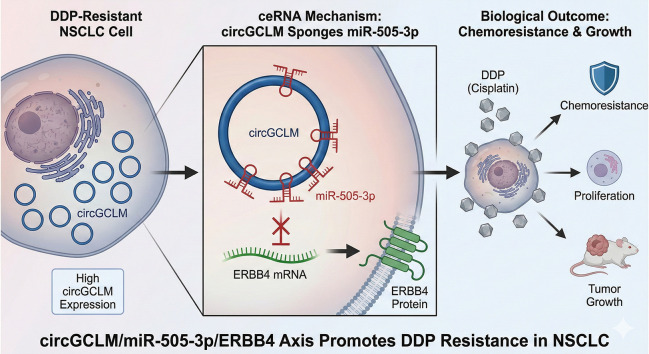

## Introduction

Globally, the burden of lung cancer is characterized by the second highest incidence among all malignancies, yet it accounts for the greatest number of cancer-related deaths [1]. NSCLC constitutes roughly 85% of lung cancers, with its histological spectrum primarily comprising adenocarcinoma (∼50%), squamous cell carcinoma (∼20-30%), and rarer entities like large cell carcinoma [2]. NSCLC has entered in a new paradigm of precision medicine, marked by low-dose CT screening to reduce mortality and the rise of targeted and immunotherapies for driver gene mutations and immune checkpoints [3]. While the therapeutic landscape for advanced NSCLC continues to evolve, DDP-based doublet regimens continue to serve as the foundational treatment for most advanced NSCLC cases [4]. Nevertheless, a major barrier to durable response is the inevitable development of DDP resistance in advanced NSCLC [5]. Consequently, a detailed understanding of DDP resistance mechanisms is critical for overcoming this therapeutic barrier and enhancing clinical prognosis.

The circular RNAs (circRNAs) constitute a conserved class of non-coding RNAs defined by a covalently closed continuous loop, which is formed via back-splicing [6,7]. This unique structure confers notable stability and often underlies their cell- or tissue-specific expression [6,7]. The covalently closed circular structure of circRNAs, resulting from a back-splicing event that links a downstream 5′ donor splice site with an upstream 3′ acceptor splice site, makes them highly resistant to RNA exonucleases, thereby providing superior stability compared to linear RNAs [8]. CircRNAs are now recognized as key regulators in cellular physiology, functioning through mechanisms including microRNA sequestration, protein interaction, transcriptional modulation, and limited translation [[9], [10], [11], [12], [13], [14]]. The aberrant expression of circRNAs is broadly implicated in various human pathologies, such as cancer, neurological disorders, and cardiovascular conditions [[15], [16], [17]]. Although studies have suggested a link between certain circRNAs and drug resistance in NSCLC, their broader functional roles and underlying mechanisms remain to be elucidated [[18], [19], [20], [21], [22]].

MicroRNAs (miRNAs) are small non-coding RNAs that function as master post-transcriptional regulators by binding to target mRNAs to induce their degradation or translational repression [23]. Through this regulatory capacity, miRNAs govern fundamental cellular processes, and their dysregulation is a hallmark of cancer that contributes to tumorigenesis, metastasis, and therapeutic resistance [24]. In the context of drug resistance, miRNAs orchestrate resistant phenotypes by modulating key pathways, such as bypass signaling, apoptotic thresholds, and drug efflux mechanisms [25]. This regulatory influence is embedded within broader ceRNA networks, where circRNAs act as molecular sponges to sequester miRNAs and prevent them from repressing their target mRNAs [26]. Consequently, the circRNA/miRNA/mRNA axis constitutes a critical post-transcriptional layer in which circRNAs indirectly modulate gene expression and therapy response by intercepting miRNAs that would otherwise silence resistance-associated genes.

The receptor tyrosine kinase ERBB4 belongs to the epidermal growth factor receptor (EGFR) family that can bind to a variety of ligands to form dimers [[27], [28], [29]]. The ERBB4 plays a context-dependent role in tumor biology, demonstrating both tumor-suppressive and oncogenic functions, where its homodimers typically induce apoptosis, growth arrest, and differentiation, while heterodimers with other ERBB receptors promote cell proliferation, migration, invasion, and chemoresistance [30]. The ERBB4 activates downstream signaling pathways including PI3K/AKT and MAPK/ERK, which are critical for cell survival and proliferation [31,32]. Accumulating evidence implicates ERBB4 in chemotherapy resistance [33]. In NSCLC, ERBB4 expression has been clinically correlated with response to platinum-based chemotherapy, with ERBB4-positive patients showing poor response to gemcitabine-cisplatin treatment [34]. However, the specific molecular mechanisms by which ERBB4 mediates DDP resistance in NSCLC, particularly its regulation by upstream non-coding RNAs such as circRNAs, remain largely unexplored.

To identify circRNAs potentially involved in DDP resistance, we first analyzed GEO datasets for differentially expressed circRNAs in NSCLC samples. Candidate circRNAs were then functionally screened using siRNA-mediated knockdown in NSCLC cells to assess their impact on DDP sensitivity. Through this systematic approach, circGCLM was identified as a top candidate significantly modulating DDP resistance and was selected for further investigation.

## Materials and methods

### Cell culture

The human NSCLC cell lines (A549, H1299), alongside 293T cells, were purchased from the American Type Culture Collection (ATCC, VA, USA). The DDP-resistant NSCLC cell lines (A549-DDP, H1299-DDP) were established through stepwise in vitro induction with DDP (Selleck, TX, USA) over a period of 6months. Concentrations were progressively increased stepwise from an initial 0.1μg/ml to a final 32μg/ml, with each concentration maintained for approximately 2–3 passages. The resistance index (RI), calculated as the ratio of IC50 values of resistant cells to parental cells, was 5 for A549-DDP and 4 for H1299-DDP, confirming successful establishment of resistant models. All cell lines were cultured with 90% RPMI-1640 added with 10% FBS and 1% penicillin/streptomycin mixture. DDP-resistant sublines were continuously cultured in medium containing 1 μg/ml DDP to maintain the resistant phenotype.

### Cell transfection

The siRNA sequences specific for hsa_circ_0003513, hsa_circ_0007798, hsa_circ_0046263, hsa_circ_0004104, hsa_circ_0014263, hsa_circ_0046533, and siRNA-NC were designed by Sangon Biotech (Shanghai, China). The shRNA lentiviral vectors for circGCLM (sh-circGCLM) knockdown and the overexpression plasmid vectors for circGCLM (OE-circGCLM) were custom-generated by Angel Biotech (Suzhou, China). The mimics of miR-505-3p (miR-505-3p-mimics), negative control mimics (miR-mimics-NC), the inhibitor of miR-505-3p (miR-505-3p-inhibitor), negative control inhibitor (miR-inhibitor-NC) and the inhibitor sponge of miR-505-3p (miR-505-3p-inhibitor sponge) were purchased from GenePharma (Shanghai, China). Corresponding shRNA or siRNA sequences are listed in Table S1.

Oligonucleotides (including siRNAs and miRNA modulators) and plasmids were transfected into cells at 80% confluency with Lipofectamine 3000 reagent (Invitrogen, CA, USA), followed by a 48-hour incubation and subsequent cell collection for further analysis. For stable gene manipulation, cells were transduced with lentivirus for 48 hours at low density. Following a 3-day expression period, selection was initiated with puromycin (1 μg/ml; Selleck, TX, USA) for one week. Successful knockdown/overexpression was confirmed via RT–qPCR.

### Cell counting kit-8 (CCK-8) assay

Cell viability was assessed using a CCK-8 kit (Dojindo, Tokyo, Japan). Briefly, NSCLC cells seeded in 96-well plates were treated with a gradient of DDP concentrations. After treatment, the cells were incubated with the CCK-8 reagent for 4 hours, and the absorbance was measured at a wavelength of 450nm.

### RT-qPCR

For RNA extraction, total RNA was isolated using TRIzol reagent (Invitrogen, CA, USA) and subsequently reverse-transcribed into cDNA using the PrimeScript RT reagent kit (Takara, Dalian, China). For subcellular localization analysis, the indicated cells were fractionated into nuclear and cytoplasmic components using the NE-PER Nuclear and Cytoplasmic Extraction Reagents (Pierce, IL, USA). The abundance of target RNAs in these fractions was then quantified by RT-qPCR using the SYBR Premix Ex Taq (Takara, Dalian, China). Gene expression was quantified using the 2−ΔΔCt method, with normalization to GAPDH or U6. Corresponding primer sequences are listed in Table S2. DNA fragments were electrophoresed on a 1% agarose gel containing nucleic acid dye. After solidification, samples were loaded and run at 200 V for 20min in 1X TAE buffer, followed by imaging with a chemiluminescence system.

### RNase R digestion assay

To assess the circular RNA stability, total RNA from A549 cells was subjected to treatment with or without RNase R (5 U/μg RNA; Selleck, TX, USA) for 30 minutes at 37°C. The purified RNA was then used for RT-qPCR analysis of circGCLM expression.

### Actinomycin D chase assay

A549 cells were exposed to actinomycin D (10μg/ml; Selleck, TX, USA) for the indicated time periods (0, 4, 8, 12, and 24 hours); total RNA was then extracted. The expression of circGCLM was subsequently analyzed by qRT-PCR.

### Edu

An Edu proliferation assay (Beyotime, Shanghai, China) was performed to measure DNA synthesis. After a 2 hours incubation with Edu to enable labeling of nascent DNA during synthesis, nuclei were counterstained with DAPI, fluorescence images were acquired using a Nikon microscope and Edu-positive cells were enumerated using ImageJ.

### Colony formation

Colony-forming ability was assessed by seeding cells (1,000 cells/well in 6-well plates) and culturing for two weeks. After fixation with paraformaldehyde and crystal violet staining, colonies containing >50 cells were counted.

### Wound healing

Confluent cells in 6-well plates were scratched with a pipette tip, washed, and then cultured in low-serum medium. Images were taken at 0, 24, and 48 hours, and the relative wound closure was quantified with ImageJ software.

### Transwell migration

A Transwell migration capability was performed using chambers with 8 μm pores. The upper chamber contained cells suspended in serum-free medium, while the lower chamber contained complete medium with 10% FBS. Following 24 hours incubation, migrated cells on the lower membrane were fixed, stained with crystal violet, and quantified microscopically in five random fields.

### Tunel assay

Cell apoptosis was detected using a Tunel staining kit (Beyotime, Shanghai, China). Cells were incubated with a mixture of TdT enzyme and Fluorescent labelling solution for 2 hours to label fragmented DNA. Nuclei were visualized with DAPI, fluorescence images were captured, and apoptotic cells were enumerated using ImageJ software.

### Flow Cytometry

Cell apoptosis was analyzed using an Annexin V-FITC apoptosis detection kit (Beyotime, Shanghai, China) with dual staining of Annexin V and PI. Cells were harvested, resuspended in 100μL binding buffer, and simultaneously stained with 10μL Annexin V and PI for 15 minutes at room temperature under light-protected conditions. Samples were acquired on a CytoFLEX flow cytometer (Beckman Coulter, CA, USA) for immediate analysis, with subsequent analysis using FlowJo software.

### RNA FISH

The subcellular localization of circGCLM was visualized by RNA FISH with a custom probe and their SA-Biotin system kit (GenePharma, Shanghai, China). Following overnight culture on coverslips, cells were fixed, permeabilized, blocked, and pre-hybridized. Pre-formed complexes of biotinylated probes and streptavidin-Cy3 were hybridized to the samples overnight at 37°C. After stringent washes to remove background, nuclei were counterstained with DAPI for confocal microscopy (Zeiss, Jena, Germany) imaging.

### Western blotting

Total cellular protein was lysed in RIPA buffer supplemented with protease and phosphatase inhibitors. Protein concentration was determined by BCA assay (Epizyme Biotech, Shanghai, China). Samples (30 μg per lane) were resolved by SDS–PAGE, transferred onto PVDF membranes, and blocked with 5% non-fat milk. Blots were probed with anti‑ERBB4 (Proteintech, China, 19943‑1‑AP) and anti‑GAPDH (Abcam, UK, ab181602) primary antibodies overnight at 4 °C, then with an HRP‑linked secondary antibody (Abcam, UK, ab205718). Detection was performed with ECL substrate using a chemiluminescence imager, and band intensities were normalized to GAPDH.

### Dual-luciferase reporter assay

To confirm direct targeting, dual‑luciferase reporter assays were conducted. Wild‑type sequences of the circGCLM or ERBB4 3′UTR harboring the predicted miR‑505‑3p binding motif were cloned into the pmirGLO vector; corresponding mutants with seed‑sequence alterations served as controls. 293T cells were co‑transfected with these reporters and either miR‑505‑3p mimics or control mimics. After 48 h, relative luciferase activity (Renilla/firefly) was determined, with normalization to firefly luciferase to account for transfection variability.

### RNA immunoprecipitation (RIP)

To detect RNA-protein interactions, we performed RNA immunoprecipitation (RIP) with the Magna RIP™ Kit (Millipore, MA, USA). Cell lysates were prepared in complete RIP lysis buffer and incubated overnight at 4 °C with magnetic beads pre-bound to 5 µg of anti‑Ago2 (CST, USA, 2897) or control IgG. Following extensive washing, immunoprecipitated complexes were digested with Proteinase K to release the bound RNAs. RNA was purified by phenol‑chloroform extraction and ethanol precipitation, then subjected to RT–qPCR. Relative enrichment of target RNAs was determined by comparing anti‑Ago2 samples to the IgG control.

### Cell line-derived xenograft (CDX) model

All animal procedures were approved by the Animal Ethics Committee of Shanghai Tongji Hospital (protocol number: 20250907-20260907-DW-1050). Female BALB/c nude mice (4-6weeks old, n=6 per group) were randomly assigned to three groups: control, circGCLM knockdown, and rescue. A549-DDP cells (5 × 10^6^) stably transfected with the indicated vectors were injected subcutaneously into the right flank. Tumor volumes were measured every 5days using digital calipers by an investigator blinded to group allocation. When tumors reached ∼100mm^3^, mice received intraperitoneal DDP (20μg/g) twice weekly. Mice were euthanized on day 30, and tumors were excised, weighed, and fixed for analysis.

### Bioinformatics

CircRNA expression analysis was performed on the GSE101586 dataset retrieved from the Gene Expression Omnibus (GEO) repository. The StarBase and TargetScan databases were used to identify putative miRNAs targeted by circGCLM and the respective mRNAs targeted by these miRNAs. Single-cell RNA sequencing data were obtained from the Gene Expression Omnibus (GEO) database under accession number GSE194070, GSE198099, GSE291670. Subsequent data analysis and presentation were carried out with R 3.6.0 and GraphPad Prism 8.0.

### Statistical analysis

All quantitative data were obtained from at least three independent experiments and were presented as the mean ± standard deviation (SD). Statistical analyses were performed using GraphPad Prism 8.0. Where indicated, two‑group comparisons were assessed by a two‑tailed Student’s t‑test. For multi‑group comparisons, one‑way analysis of variance (ANOVA) was used, with Tukey’s honest significant difference test for post‑hoc analysis. In all cases, P < 0.05 was taken as the threshold for statistical significance.

## Results

### Identification and characterization of circGCLM in NSCLC

**Fig. 1:**
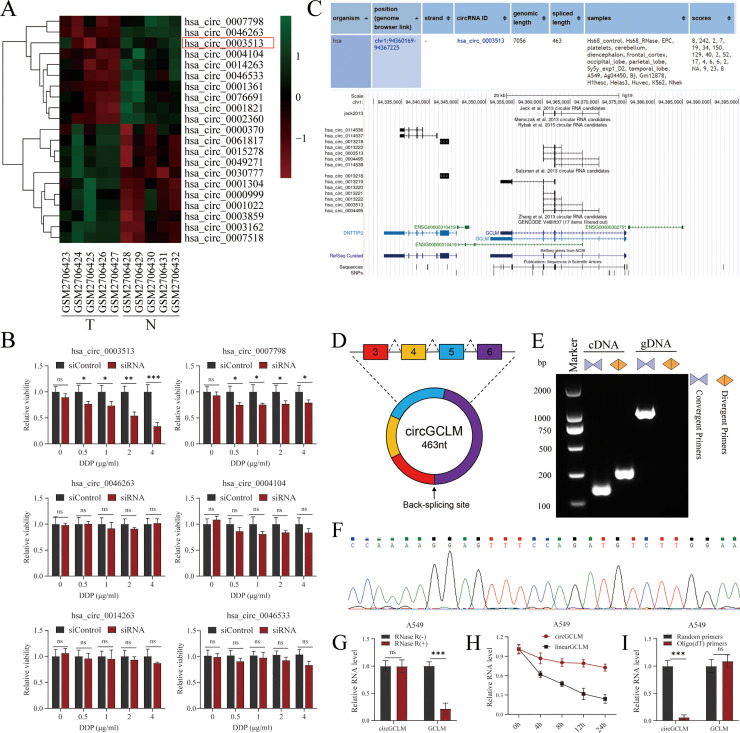
Identification and characterization of circGCLM in NSCLC. (A) Heatmap showing differentially expressed circRNAs between NSCLC tumor tissues (T, n=5) and adjacent normal tissues (N, n=5) from GSE101586 dataset. (B) CircGCLM knockdown resulted in the most significant reduction in cell viability. (C) Genomic locus of circGCLM (hsa_circ_0003513) derived from the GCLM gene. (D) Schematic illustration of circGCLM formation via back-splicing of exons 3-6. (E) Validation of circGCLM back-splice junction. Divergent primers amplified circGCLM from cDNA but not from gDNA. (F) Sanger sequencing confirming the back-splice junction site. (G) CircGCLM was resistant to RNase R digestion, while linear GCLM was significantly degraded. (H) Half-life of circGCLM and linear GCLM was measured by transcript inhibition assay. (I) RT-qPCR using random vs. oligo(dT) primers confirming the absence of a poly(A) tail on circGCLM. Data in (B, G-I) are mean ± SD from three independent experiments. *P<0.05, **P<0.01, ***P<0.001 by Student's t-test or one-way ANOVA with Tukey's post hoc test.

A bioinformatic screening was performed to pinpoint circRNAs associated with NSCLC pathogenesis and chemoresistance. We interrogated the GEO dataset GSE101586, which contains circRNA expression data from five NSCLC tumor samples and five matched normal controls (Fig. 1A). CircRNAs meeting the thresholds of adjusted P < 0.05 and |log₂(fold change)| > 2 were classified as differentially expressed. Following the bioinformatic identification of significantly dysregulated circRNAs, we sought to determine their functional role in DDP chemosensitivity. We selected the top six most significantly downregulated candidates for functional validation. Each candidate was knocked down in A549 cells using specific siRNAs and a scrambled siRNA was included as a control. To assess the impact on DDP sensitivity, we treated the siRNA-transfected cells with a gradient of DDP concentrations and measured cell viability using the CCK-8 assay. Notably, the knockdown of hsa_circ_0003513 (termed circGCLM) resulted in a significant increase in sensitivity to DDP (Fig. 1B). These findings suggested that circGCLM might be a specific key regulator of DDP responsiveness and that its dysregulation might lead to DDP resistance in NSCLC. The circBase database indicated that circGCLM is a circular transcript originating from its parent gene GCLM locus, produced via a back-splicing event that joins exon 3 directly to exon 6 (Fig. 1C-D). To experimentally confirm its circular nature and the precise back-splicing junction, we designed divergent primers spanning the predicted junction site. CircGCLM was amplified by divergent primers from cDNA but not from genomic DNA (gDNA) (Fig. 1E), and Sanger sequencing of the product confirmed its back‑splicing junction (Fig. 1F). Further biochemical validation supported its circular conformation: CircGCLM resisted RNase R digestion, while the mRNA level of its linear host gene was significantly degraded (Fig. 1G). Transcriptional inhibition assays with actinomycin D revealed that circGCLM possesses a significantly longer half-life than its linear counterpart (Fig. 1H). Oligo(dT) enrichment assays confirmed the absence of a poly(A) tail on circGCLM (Fig. 1I). These results demonstrated the existence and specific structure of circGCLM in our experimental system.

### CircGCLM was upregulated in DDP-resistant NSCLC cells and modulated DDP sensitivity

**Fig. 2:**
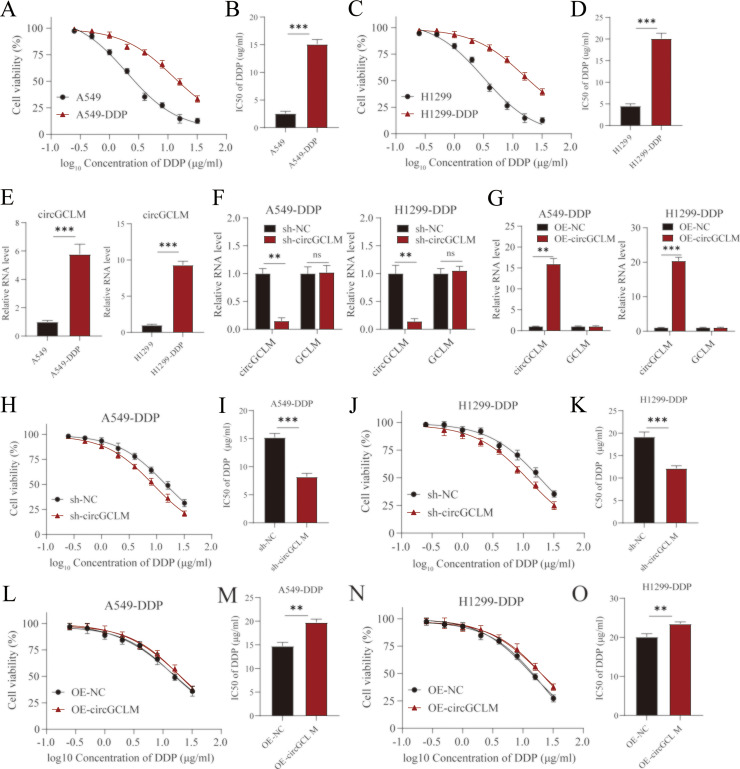
CircGCLM was upregulated in DDP-resistant NSCLC cells and modulated DDP sensitivity. (A) A549 and A549-DDP cells were treated with indicated concentrations of DDP for 48 h, and cell viability was assessed by CCK-8 assay. (B) IC50 values of DDP in A549 vs. A549-DDP cells. (C-D) Same as (A-B) for H1299 and H1299-DDP cells. (E) RT-qPCR analysis of circGCLM expression in parental vs. DDP-resistant cells. (F-G) Validation of circGCLM knockdown (F) and overexpression (G) efficiency by RT-qPCR. (H-I) Effect of circGCLM knockdown on DDP sensitivity. Cell viability curves (H) and IC50 (I) values in A549-DDP cells transfected with sh-NC or sh-circGCLM. (J-K) Same as (H-I) for H1299-DDP cells. (L-M) Effect of circGCLM overexpression on DDP sensitivity. Cell viability curves (L) and IC50 (M) values in A549-DDP cells transfected with vector or circGCLM overexpression plasmid. (N-O) Same as (L-M) for H1299-DDP cells. All data are mean ± SD from three independent experiments. **P<0.01, ***P<0.001 by Student's t-test or one-way ANOVA with Tukey's post hoc test.

To functionally characterize circGCLM in acquired DDP resistance, we derived resistant counterparts from parental NSCLC lines by chronically exposing parental cells to escalating doses of DDP [35]. The resulting resistant cells exhibited a significantly higher IC50 for DDP than their parental counterparts (Fig. 2A-D). Importantly, we found that the circGCLM expression was also substantially elevated in the DDP-resistant cells (Fig. 2E), suggesting a potential link between circGCLM and the DDP-resistant phenotype. To directly test this hypothesis, we knocked down circGCLM in the DDP-resistant cells using specific shRNAs. Depletion of circGCLM (Fig. 2F) significantly sensitized the resistant cells to DDP, as demonstrated by a substantial decrease in both cellular viability and IC50 in CCK-8 assays following DDP treatment (Figs. 2H-K). Conversely, overexpression of circGCLM (Fig. 2G) in the cells was sufficient to confer resistance, leading to enhanced cell survival upon DDP exposure and a significant increase in the IC50 (Figs. 2 L-O).

### CircGCLM absence suppressed proliferation and migration and induced apoptosis in DDP-resistant NSCLC cells

**Fig. 3:**
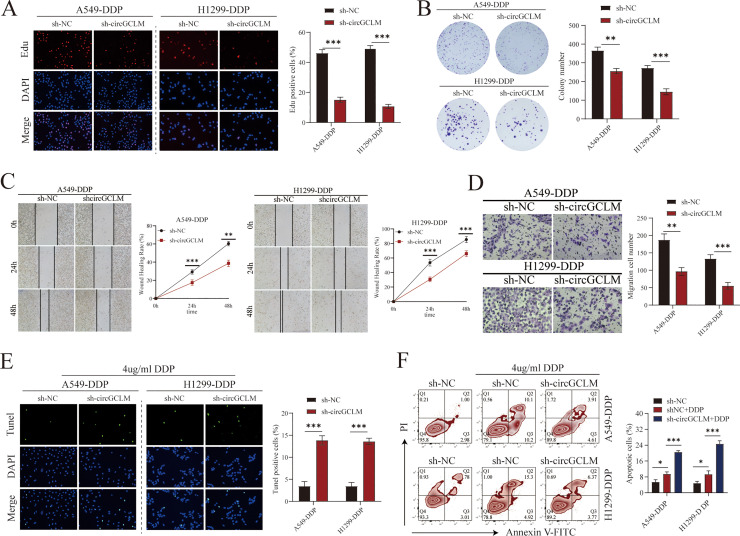
CircGCLM absence suppressed proliferation and migration and induced apoptosis in DDP-resistant NSCLC cells. (A) Edu incorporation assay. Representative images (left) show Edu-positive proliferating cells (red); nuclei were counterstained with DAPI (blue). Scale bar, 50μm. Quantification of Edu-positive cells (right). (B) Colony formation assay. Representative images of crystal violet-stained colonies (left) and quantification of colony numbers (right). (C) Wound healing assay. Representative images at 0, 24, and 48 h post-scratch (left) and quantification of wound closure rate (right). Scale bar, 100μm. (D) Transwell migration assay. Representative images of migrated cells stained with crystal violet (left) and quantification of migrated cell numbers per field (right). Scale bar, 100μm. (E) Tunel assay. Representative images (left) showing Tunel-positive apoptotic cells (green) and DAPI-stained nuclei (blue). Scale bar, 50μm. Quantification of Tunel-positive cells (right). (F) Flow cytometry analysis of apoptosis by Annexin V-FITC/PI staining. Representative plots (left) and quantification of total apoptotic rate (right). All data are mean ± SD from three independent experiments. **P<0.01, ***P<0.001 by Student's t-test or one-way ANOVA with Tukey's post hoc test.

The phenotypic impact of circGCLM knockdown was evaluated through a series of in vitro functional assays. Depletion of circGCLM significantly inhibited the cell proliferation. This was demonstrated by a pronounced decline in the percentage of cells undergoing active DNA synthesis (Fig. 3A) and a substantial decrease in colony-forming ability in long-term clonogenic assays (Fig. 3B), relative to the control group. Furthermore, the migratory capacity of the cells was severely compromised upon circGCLM knockdown. Migration was markedly attenuated upon circGCLM depletion, with significant reductions in both wound‑closure distance and the number of cells traversing transwell membranes. (Figs. 3C-D). In addition, loss of circGCLM promoted apoptosis, as indicated by increased tunel-positive staining (Fig. 3E) and elevated apoptotic rates measured by flow cytometry (Fig. 3F). Taken together, these findings established circGCLM as a promoter of NSCLC tumorigenesis and progression.

### CircGCLM functioned as a ceRNA by sponging miR-505-3p to regulate ERBB4 expression

**Fig. 4:**
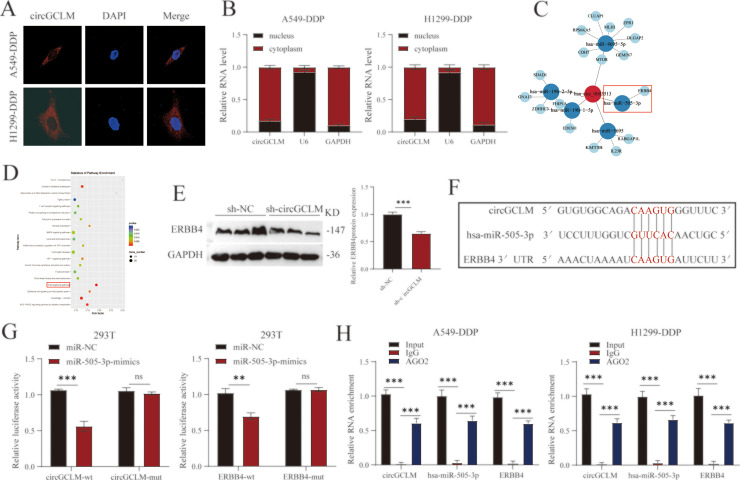
CircGCLM functioned as a ceRNA by sponging miR-505-3p to regulate ERBB4 expression. (A) RNA-FISH localization of circGCLM (red) in DDP-resistant cells. Nuclei were stained with DAPI (blue). Scale bar, 10 μm. (B) RT-qPCR analysis of circGCLM in nuclear and cytoplasmic fractions. U6 (nuclear control) and GAPDH (cytoplasmic control) were used as fraction-specific markers. (C) Predicted ceRNA network of circGCLM-miRNA-mRNA interactions based on starBase and TargetScan databases. (D) KEGG pathway enrichment analysis of predicted target genes, highlighting the ERBB signaling pathway. (E) Western blot analysis of ERBB4 protein expression in DDP-resistant cells transfected with sh-NC or sh-circGCLM. GAPDH served as loading control. (F) Putative miR-505-3p binding sites in circGCLM and the ERBB4 3′UTR. (G) Dual-luciferase reporter assays in 293T cells co-transfected with indicated reporters and miR-505-3p mimics or control mimics. Firefly luciferase activity was normalized to Renilla. (H) RIP assays using anti-Ago2 or control IgG in DDP-resistant cell lysates. Enrichment of circGCLM, miR-505-3p, and ERBB4 mRNA in Ago2 immunoprecipitates was analyzed by RT-qPCR and expressed as fold enrichment relative to IgG control. All data are mean ± SD from three independent experiments. **P<0.01, ***P<0.001 by Student's t-test or one-way ANOVA with Tukey's post hoc test.

A classic and extensively studied mode of action for circRNAs is to function as ceRNAs by sponging miRNAs to counteract miRNA-mediated silencing and regulate target gene expression [36]. To explore the potential role of circGCLM in our context, we first assessed the subcellular distribution of circGCLM by RNA FISH and cellular fractionation. Both methods demonstrated its predominant localization in the cytoplasm (Fig. 4A-B), suggesting its potential function as a ceRNA. Bioinformatic prediction using starBase and TargetScan databases identified several candidate miRNAs that may bind circGCLM and the respective mRNAs targeted by these miRNAs (Fig. 4C). Intersection with enrichment analysis of related pathways (Fig. 4D) highlighted the ERBB signaling pathway and a key mRNA, ERBB4, as potential downstream effectors. Notably, depletion of circGCLM led to a pronounced downregulation of ERBB4 at the protein level (Fig. 4E), supporting a possible regulatory axis. Bioinformatics analyses revealed complementary sequences between circGCLM and miR‑505‑3p, and between miR‑505‑3p and the ERBB4 3′‑UTR (Fig. 4F). In dual‑luciferase assays, miR‑505‑3p mimics suppressed the activity of wild‑type (WT) reporter constructs containing these sequences, an effect abolished by mutation of the binding sites (Fig. 4G). RIP with an anti‑Ago2 confirmed that circGCLM, miR‑505‑3p, and ERBB4 mRNA coexist within the complex (Fig. 4H). Together, these data demonstrated that circGCLM functioned as a molecular sponge, sequestering miR‑505‑3p to derepress ERBB4 expression.

### Inhibition of miR-505-3p reversed the tumor-suppressive effects of circGCLM knockdown in vitro and in vivo

**Fig. 5:**
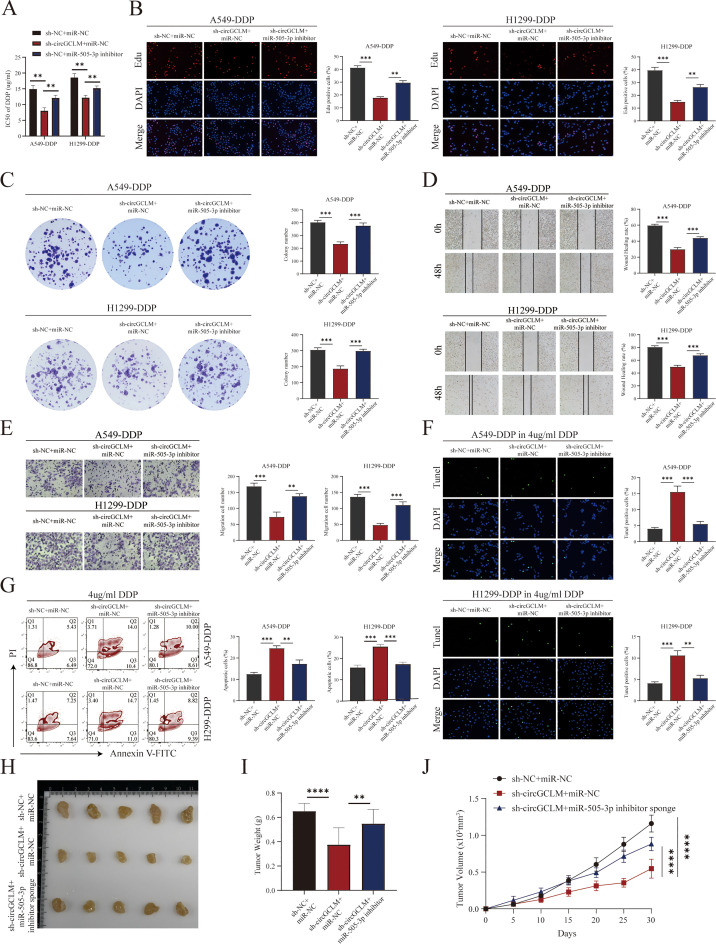
Inhibition of miR-505-3p reversed the tumor-suppressive effects of circGCLM knockdown in vitro and in vivo. (A) IC50 values of DDP determined by CCK-8 assay. (B) Edu incorporation assay. Quantification of Edu-positive cells. (C) Colony formation assay. Quantification of colony numbers. (D) Wound healing assay. Quantification of wound closure rate at 48 h. (E) Transwell migration assay. Quantification of migrated cells per field. (F) Tunel assay. Quantification of Tunel-positive cells. (G) Flow cytometry analysis. Quantification of total apoptotic rate. (H) Tumor images at endpoint (day 30). (I) Tumor weights at endpoint. (J) Tumor volume measured every 5 days. All data are mean ± SD from three independent experiments. **P<0.01, ***P<0.001, ****P<0.0001 by Student's t-test or one-way ANOVA with Tukey's post hoc test.

To delineate the functional axis between circGCLM and miR-505-3p, we performed comprehensive phenotypic rescue assays across three groups: a negative control (sh-NC+miR-NC), a circGCLM knockdown group (sh-circGCLM+miR-NC), and a rescue group with concurrent circGCLM knockdown and miR-505-3p inhibition (sh-circGCLM + miR-505-3p inhibitor). Knockdown of circGCLM significantly sensitized NSCLC cells to DDP, as evidenced by a decreased IC50 compared to the negative control. This sensitization effect was substantially reversed upon concurrent inhibition of miR-505-3P (Fig. 5A), suggesting that circGCLM promoted DDP resistance primarily by sequestering miR-505-3p. The anti-proliferative effect caused by circGCLM knockdown, demonstrated by reduced Edu incorporation and diminished colony-forming capacity, was markedly rescued by co-transfection with miR-505-3p inhibitor (Fig. 5B-C). Similarly, the impaired cell migration observed in wound healing and transwell assays following circGCLM depletion was largely restored upon miR-505-3p inhibition (Fig. 5D-E). Finally, the increased apoptosis induced by circGCLM knockdown, quantified by Tunel staining and flow cytometry, was effectively attenuated by miR-505-3p (Fig. 5F-G). Collectively, these results demonstrated that the oncogenic phenotypes mediated by circGCLM were dependent on its functional sponging of miR-505-3p. To evaluate the function of circGCLM in vivo, we established subcutaneous xenografts using DDP‑resistant NSCLC cells. Tumors in the circGCLM‑knockdown group exhibited slower growth than those in the control group (Fig. 5H). At the experimental endpoint, both tumor weight and volume were reduced upon circGCLM depletion (Fig. 5I-J). Crucially, the antitumor effect of circGCLM knockdown was substantially reversed by concurrent miR-505-3p inhibition, providing direct in vivo evidence that the oncogenic function of circGCLM was mediated through sponging miR-505-3p.

### Visualization of malignant epithelial cells from integrated scRNA-seq datasets revealed ERBB4 expression dynamics in treated and untreated groups

**Fig. 6:**
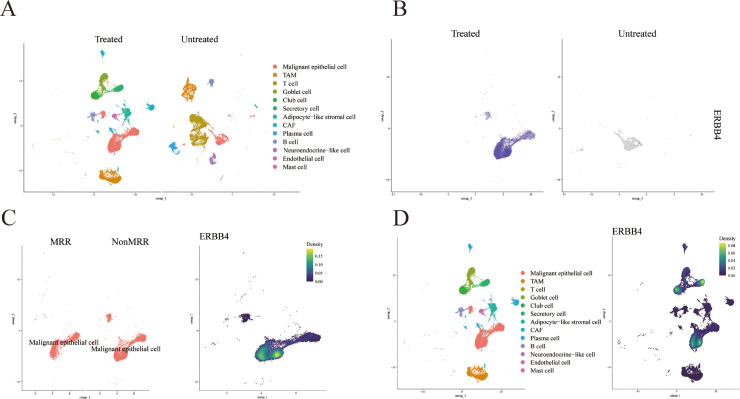
Visualization of malignant epithelial cells from integrated scRNA-seq datasets revealed ERBB4 expression dynamics in treated and untreated groups. (A) UMAP of the integrated GSE194070, GSE198099 (Untreated), and GSE291670 (Treated) datasets. (B) Feature plot highlighting ERBB4 expression across the integrated dataset. ERBB4 expression is elevated in the Treated group compared to the Untreated cohort in malignant epithelial cell. (C) UMAP of the GSE291670 (Treated) cohort only, split by pathological response: Major Pathologic Response (MPR) and Non-Major Pathologic Response (NonMPR), demonstrating higher ERBB4 expression in the NonMPR group. (D) Within the GSE291670 treated cohort, a feature plot comparing ERBB4 expression between normal epithelial cells and malignant epithelial cells, showing higher expression in the normal epithelial cells following treatment.

To investigate the transcriptional landscape of NSCLC in the context of therapeutic response, we integrated single-cell RNA sequencing data from the GSE194070, GSE198099 (Untreated), and GSE291670 (Treated, Neoadjuvant anlotinib combined with PD-1 blockade therapy) datasets. UMAP visualization revealed distinct clustering patterns associated with treatment status (Fig. 6A). Feature plot analysis demonstrated that ERBB4 expression was elevated in the Treated group compared to the Untreated cohort, suggesting that ERBB4 expression may be induced or selected for upon therapeutic exposure (Fig. 6B). Within the GSE291670 Treated cohort, we further stratified patients based on pathological response. UMAP projection of the treated cohort showed that ERBB4 was higher in the NonMPR subgroup relative to the MPR group, indicating an association with therapeutic resistance (Fig. 6C). Interestingly, in the GSE291670 treated cohort, we observed that normal epithelial cells exhibited higher ERBB4 expression compared to their malignant epithelial cell counterparts following treatment (Fig. 6D). This differential expression pattern suggests that while malignant cells upregulate ERBB4 in response to treatment, this upregulation remains below the level observed in normal cells, potentially reflecting a differential dependency or adaptive mechanism. These findings identify ERBB4 as a potential marker of treatment exposure and resistance, with differential upregulation between normal and malignant epithelial cells highlighting the complex transcriptional heterogeneity underlying therapeutic response.

## Discussion

CircRNAs are a class of endogenous non-coding RNAs defined by a covalently closed continuous loop, exerting widespread regulatory effects on tumor progression, metastasis, and therapy, largely via functioning as ceRNAs for miRNAs or by interacting directly with proteins [8,9,37]. Their inherent stability and frequent dysregulation in malignancies position them as diagnostic biomarkers and targets for therapeutic intervention [[38], [39], [40], [41]]. We characterized circGCLM, a circular RNA originating from the GCLM locus (Fig. 1D), whose expression was significantly altered in NSCLC and correlated with resistance to DDP.

Our work began with a systematic bioinformatic screening, which pinpointed circGCLM as a candidate based on its marked dysregulation in NSCLC datasets, and subsequent functional validation via siRNA knockdown confirmed its pivotal role in DDP resistance. While this screening approach was based on tumorigenesis rather than direct drug resistance, it rested on the rationale that tumor-initiating properties often overlapped with mechanisms underlying chemoresistance. Accordingly, circRNAs dysregulated in tumor tissues might represent candidates with potential dual roles in both tumor progression and drug resistance. We acknowledged this as an initial screening strategy that identified candidates for subsequent functional validation specifically in the context of DDP resistance. Subsequent experimental validation confirmed its circular nature including back-splicing junction sequencing and RNase R resistance. Upon generating DDP‑resistant A549 and H1299 sublines, we observed a pronounced elevation of circGCLM expression. Functional loss-of-function studies demonstrated that silencing circGCLM significantly sensitized cells to DDP, concurrently inhibiting proliferation, migration, and enhancing apoptosis. This positioned circGCLM as a central oncogenic driver contributing to the aggressive and therapy-resistant phenotype of NSCLC, aligning with the growing recognition of circRNAs as key mediators of chemoresistance in various cancers [[42], [43], [44]].

A predominant mechanism through which circRNAs exert their regulatory roles is by acting as miRNA sponges, a function embedded within the ceRNA [[45], [46], [47], [48]]. By competitively binding to miRNAs, circRNAs prevent these miRNAs from interacting with their cognate messenger RNAs, thus modulating the expression of downstream genes in a precise manner [49]. leveraging the ceRNA hypothesis, We utilized bioinformatics tools to predict miRNAs that could be sponged by the circGCLM, along with their target mRNAs, and subsequently integrated these interactions to construct a putative regulatory network (Fig. 4 C). Through this sponging activity, circGCLM derepressed the expression of ERBB4. The role of ERBB4 is highly context-dependent, exhibiting considerable plasticity rather than a strictly oncogenic or tumor-suppressive function; its activity is dynamically modulated by cell type, signaling milieu, and disease stage, often culminating in potent oncogenic driver effects in specific cancer settings [50]. A series of rescue experiments provided compelling evidence for this axis: the phenotypic consequences of circGCLM knockdown that increased DDP sensitivity and attenuated malignant behaviors were effectively reversed upon co-inhibition of miR-505-3p. We also found that ERBB4 protein expression was downregulated following circGCLM knockdown. The specific molecular interactions among circGCLM, miR-505-3p, and ERBB4 were experimentally confirmed. These confirmed that the oncogenic functions of circGCLM were largely dependent on its regulation of the miR-505-3p/ERBB4 pathway. Finally, the relevance of our findings was substantiated in vivo. In a NSCLC xenograft model, knockdown of circGCLM potently inhibited tumor growth and increased the effectiveness of DDP treatment., mirroring our in vitro observations.

Ongoing discovery and functional analysis of circRNAs are fundamentally advancing the knowledge of post-transcriptional regulatory networks in cancer biology [51,52]. The novelty of our work lay in the identification of the specific molecular triad: circGCLM/miR-505-3p/ERBB4. While dysregulation of ERBB4 signaling has been implicated in various cancers, its upstream regulation by a circRNA-mediated ceRNA network contributing to DDP resistance in NSCLC was, to our knowledge, reported here for the first time. The choice of ERBB4 as the terminal effector of this axis was of particular biological significance. ERBB4, a member of the EGFR family, is a transmembrane receptor with complex, context-dependent roles in cancer [50]. In our model of NSCLC, the circGCLM-induced upregulation of ERBB4 likely activates downstream pro-survival and proliferative pathways. Future studies aimed at mapping the specific phosphorylated tyrosine residues and interacting partners upon ERBB4 activation in this context will further refine the mechanistic model. We acknowledge several limitations in our current work. While we have established a core ceRNA axis, the complexity of RNA regulatory networks suggests that circGCLM might sponge additional miRNAs or even interact with RNA-binding proteins, contributing to its full functional repertoire. Furthermore, the upstream regulatory mechanisms controlling the biogenesis of circGCLM itself remain unknown. Identifying the host gene splicing factors or cis-acting elements that modulate its circularization could reveal new points for intervention.

Based on our previous findings, we further leveraged integrated single-cell transcriptomic analysis to uncover the dynamics of ERBB4 expression in the context of NSCLC and its association with therapeutic response. Our findings demonstrate that ERBB4 expression is significantly elevated in treated tumors compared to untreated cohorts, with further enrichment observed in the NonMPR subgroup. These observations position ERBB4 as a putative marker of treatment exposure and a potential driver of therapeutic resistance. While ERBB4has traditionally been studied in the context of developmental biology and tumorigenesis, its role in modulating therapeutic outcomes is less well characterized. Our data suggest that ERBB4 upregulation may represent an adaptive survival mechanism employed by malignant cells under therapeutic pressure. Intriguingly, we also observed that normal cells maintained higher ERBB4 expression than their malignant counterparts following treatment, suggesting that malignant cells may retain a degree of ERBB4 regulation but fail to achieve the levels observed in normal epithelium. Extrapolating from our findings, we hypothesize that ERBB4 may play a broader role in mediating resistance to multiple therapeutic modalities, including DDP-based chemotherapy.

However, several limitations of this study should be acknowledged. Although we established the circGCLM/miR-505-3p/ERBB4 regulatory axis in vitro, validation using clinical tissue samples from NSCLC patients with known DDP response status is lacking and represents an important direction for future investigation. Furthermore, while we identified ERBB4 as a downstream target, we did not experimentally examine the activation status of its probable downstream signaling pathways in response to circGCLM modulation. Addressing these limitations in future studies will further elucidate the role of circGCLM in NSCLC chemoresistance.

## Ethics approval and consent to participate

The Animal Ethics Committee of Shanghai Tongji Hospital reviewed and approved all animal procedures (protocol number 20250907-20260907‑DW‑1050). Experiments were conducted in compliance with institutional and national guidelines for the care and use of laboratory animals.

## Consent for publication

Not applicable.

## Availability of data and materials

The data supporting the findings of this study are available within the article. Additional data are available from the corresponding author upon reasonable request.

## Funding

Research reported in this publication was funded by the National Key Research and Development Program of China (No. 2023YFC2508604), the National Natural Science Foundation of China (Nos. 82273417, and 32470604), and the Shanghai Health Leading Talent Project (No. 2022LJ004).

## CRediT authorship contribution statement

**Yirou Ma:** Writing – original draft. **Baiqing Huang:** Investigation. **Haiyang Wang:** Investigation. **Mengchao Xue:** Formal analysis. **Yiyang Liu:** Investigation. **Yibo Jin:** Investigation. **Chunping Wang:** Investigation. **Shaorui Gu:** Conceptualization. **Yongxin Zhou:** Conceptualization.

## Declaration of competing interest

The authors declare that they have no known competing financial interests or personal relationships that could have appeared to influence the work reported in this paper.

## Appendix Supplementary materials

Image, application 1

Image, application 2

Image, image 3 dummy alt text

Image, image 4 dummy alt text
