# Supplementary material for: Single-cell transcriptomic analysis reveals that the circRNA circGCLM promotes tumorigenesis and confers cisplatin resistance in NSCLC through the miR-505-3p/ERBB4 axis
Source: Transl Oncol. 2026 Apr 7;67:102759. doi: 10.1016/j.tranon.2026.102759 (PMC13090960; doi:10.1016/j.tranon.2026.102759)
Supplement: Supplementary file 1 [file mmc1.docx]

Table S1

| Gene | Target | Sequence (5'→3') |
| --- | --- | --- |
| circGCLM | shRNA | CAAAAGGAGTTTCCAGATGTC |
| miR-505-3p | mimics | GGGAGCCAGGAAGUAUUGAUGU |
|  | inhibitor | ACAUCAAUACUUCCUGGCUCCC |

Table S2

| Gene | Primer | Primer Sequence (5'→3') |
| --- | --- | --- |
| circGCLM | Forward | GGCACAGGTAAAACCAAATAGTAAC |
|  | Reverse | CATGAGATACAGTGCATTCCAAGA |
| GCLM | Forward | AAATGAAAGTTTCTGCAAAACTGTT |
|  | Reverse | TCCCAGTAAGGCTGTAAATGCTC |
| miR-505-3p | RT | GTCGTATCCAGTGCAGGGTCCGAGGTATTCGCACTGGA  TACGACAGGAAA |
|  | Forward | CGCGTCAACACTTGCTGG |
|  | Reverse | AGTGCAGGGTCCGAGGTATT |
| ERBB4 | Forward | TGGTGGGCTCTTCATTCTGG |
|  | Reverse | TGAGCTTGATTGGGTGCTGT |
| GAPDH | Forward | GGCCTCCAAGGAGTAAGACC |
|  | Reverse | AGGGGAGATTCAGTGTGGTG |
| U6 | RT | GTCGTATCCAGTGCAGGGTCCGAGGTATTCGCACTGGA  TACGACAAAATA |
|  | Forward | AGAGAAGATTAGCATGGCCCCTG |
|  | Reverse | CAGTGCAGGGTCCGAGGTA |
