# Supplementary material for: Single-cell transcriptomic analysis reveals that the circRNA circGCLM promotes tumorigenesis and confers cisplatin resistance in NSCLC through the miR-505-3p/ERBB4 axis
Source: Transl Oncol. 2026 Apr 7;67:102759. doi: 10.1016/j.tranon.2026.102759 (PMC13090960; doi:10.1016/j.tranon.2026.102759)
Supplement: Supplementary file 2 [file mmc2.pdf]

Tongji Hospital Affiliated to Tongji University (Shanghai Tongji Hospital)

Ethical Review Opinion on Laboratory Animal Welfare for Research Project Applications

Reference Number	2025-DWZX-0105-001		
Project Name	Study on the mechanism of circGCLM promoting Non-Small Cell Lung Cancer progression and cisplatin resistance		
Research Institution	Tongji Hospital Affiliated to Tongji University		
Principal Investigator	Yongxin Zhou	Department	Thoracic Surgery
Signature of the Applicant	Date：2025/9/9		
Research Animals and Sources	1. Animal Type： BALB/c nude Mouse<br>2. Animal Source： Suzhou Saiye and Shanghai JieSJ Experimental Animal Co., Ltd)<br>3. The specific experimental protocol has been uploaded		

Review date	2025/9/12	Review location	Office of the Animal Ethics Committee
Research Office Opinion	The research proposal submitted is well-designed, demonstrating both scientific and societal value. The research design takes into account the protection, welfare, and ethical considerations of laboratory animals. Approval is hereby granted.<br>Research Office (Seal)<br>Date：2025/9/12		
Animal Ethics Committee Opinion	The research proposal submitted is well-designed, taking full account of minimising the number of experimental animals and sampling frequency to ensure reduced animal distress. It complies with principles of animal protection, welfare and ethics, and has been duly considered.<br>Animal Ethics Committee (Seal)<br>Date：2025/9/12		
