# Supplementary figures and images for: Single-cell transcriptomic analysis reveals that the circRNA circGCLM promotes tumorigenesis and confers cisplatin resistance in NSCLC through the miR-505-3p/ERBB4 axis
Source: Transl Oncol. 2026 Apr 7;67:102759. doi: 10.1016/j.tranon.2026.102759 (PMC13090960; doi:10.1016/j.tranon.2026.102759)

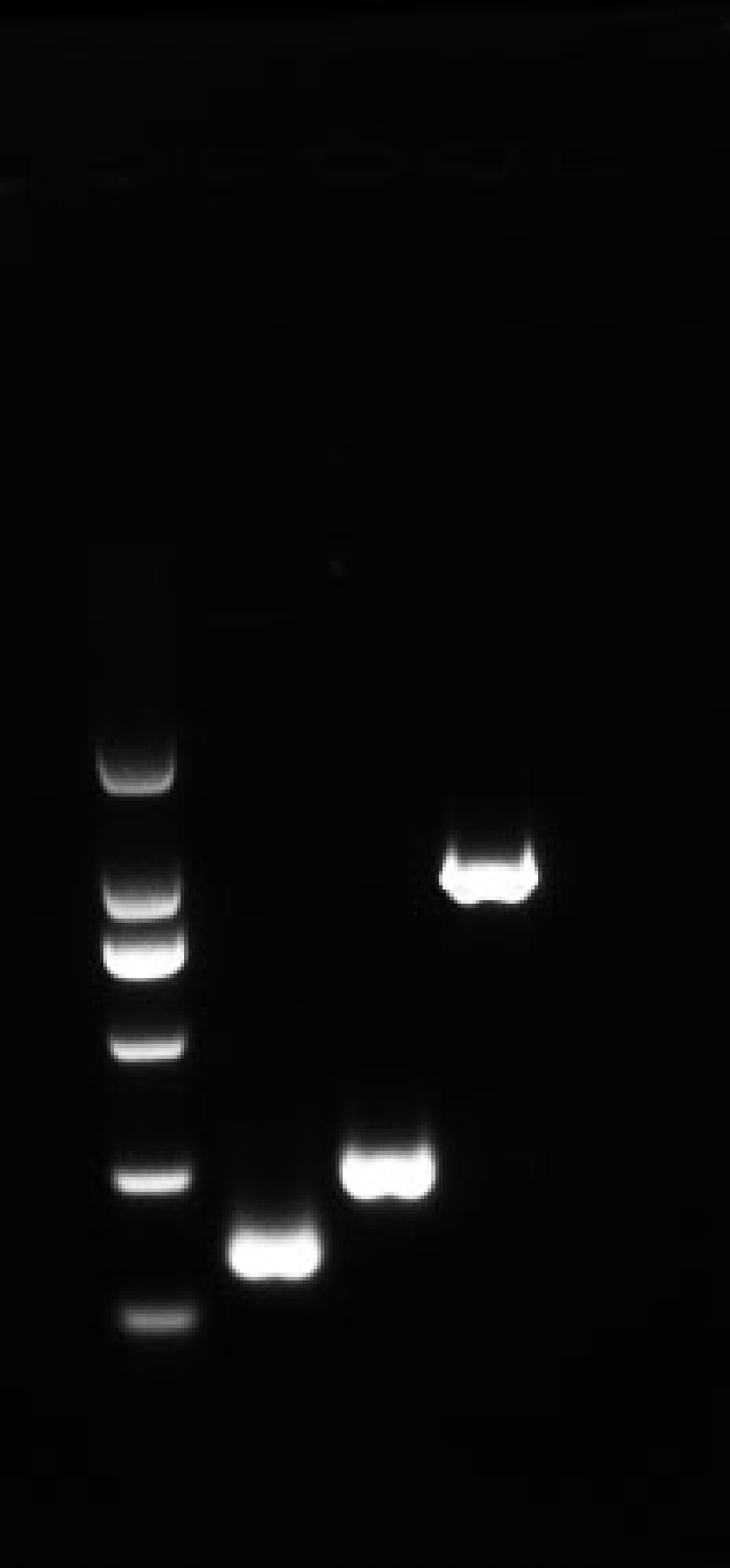

Supplement: Supplementary file 3 [file mmc3.jpg]

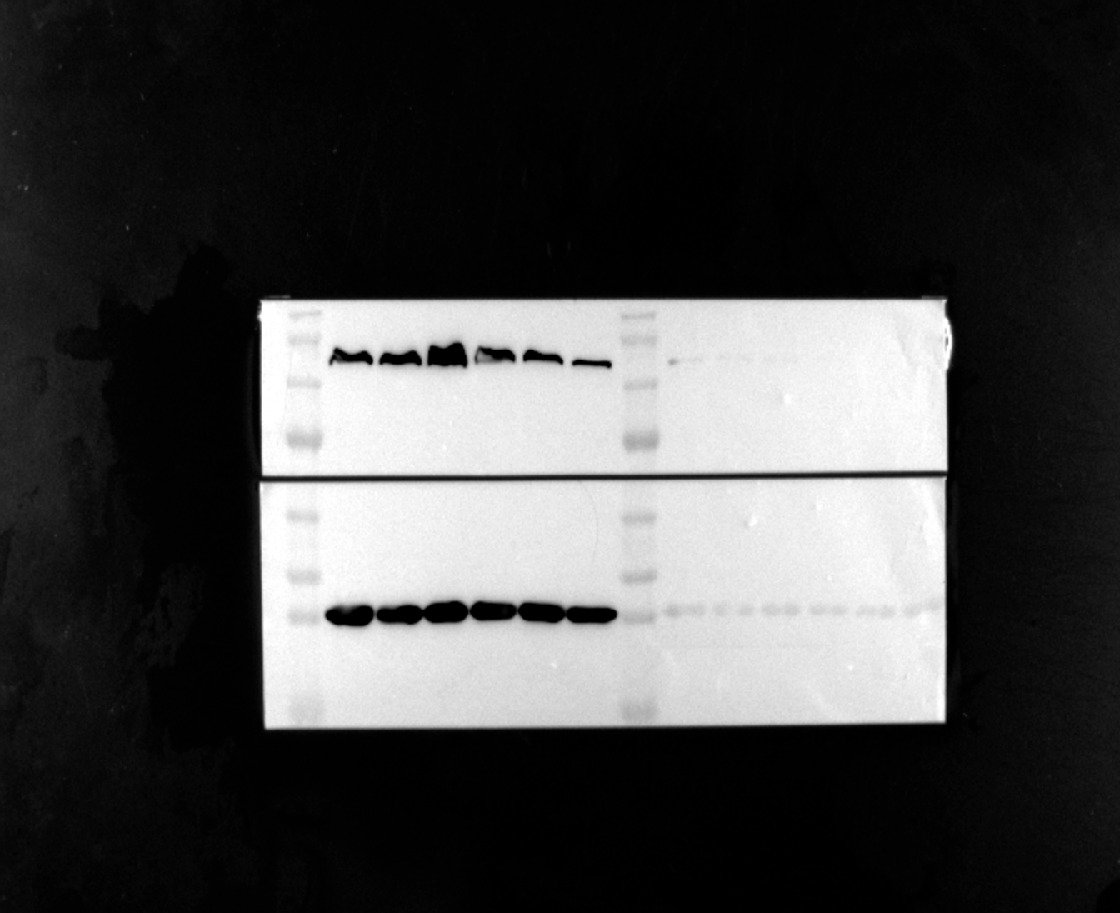

Supplement: Supplementary file 4 [file mmc4.jpg]
